# Roles of hepatic stellate cells in NAFLD: From the perspective of inflammation and fibrosis

**DOI:** 10.3389/fphar.2022.958428

**Published:** 2022-10-13

**Authors:** Man Wang, Lei Li, Yannan Xu, Juan Du, Changquan Ling

**Affiliations:** ^1^ School of Traditional Chinese Medicine, Naval Medical University, Shanghai, China; ^2^ Department of Emergency, Changhai Hospital, Naval Medical University, Shanghai, China

**Keywords:** nonalcoholic fatty liver disease, nonalcoholic steatohepatitis, hepatic stellate cells, inflammation, fibrosis

## Abstract

Non-alcoholic fatty liver disease (NAFLD) has become one of the most common diseases and severe problems worldwide because of the global increase in obesity, dyslipidemia, hypertension, and type 2 diabetes mellitus. NAFLD includes a wide spectrum of liver diseases, the histological forms of which range from non-alcoholic fatty liver (NAFL), which is generally nonprogressive, to non-alcoholic steatohepatitis (NASH), which can progress to chronic hepatitis, liver cirrhosis (LC), and sometimes hepatocellular carcinoma (HCC). Unlike NAFL, as the progressive form of NAFLD, NASH is characterized by the presence of inflammation with or without fibrosis in addition to hepatic steatosis. Although it is widely known and proved that persistent hepatic injury and chronic inflammation in the liver activate quiescent hepatic stellate cells (HSCs) and lead to hepatic fibrosis, the three-step process of “inflammation-fibrosis-carcinoma” in NAFLD has not been investigated and clarified clearly. In this process, the initiation of inflammation in the liver and the function of various liver inflammatory cells have been discussed regularly, while the activated HSCs, which constitute the principal cells responsible for fibrosis and their cross-talk with inflammation, seem not to be investigated specifically and frequently. Also, accumulated evidence suggests that HSCs can not only be activated by inflammation but also participate in the regulation of liver inflammation. Therefore, it is necessary to investigate the unique roles of HSCs in NAFLD from the perspective of inflammation and fibrosis. Here, we review the pivotal effects and mechanisms of HSCs and highlight the potential value of HSC-targeted treatment methods in NAFLD.

## Introduction

Non-alcoholic fatty liver disease (NAFLD), including two subtypes of non-alcoholic fatty liver (NAFL) and non-alcoholic steatohepatitis (NASH), is increasingly prevalent and represents a growing challenge in terms of prevention and treatment (Chalasani et al., 2018). For defining NAFLD, there must be evidence of hepatic steatosis, either by imaging or histology, and lack of secondary causes of hepatic fat accumulation such as significant alcohol consumption, long-term use of a steatogenic medication, or monogenic hereditary disorders (Tsujisaki et al., 2022). NAFL is a nonprogressive form of NAFLD in which fat accumulates in the liver but little or no inflammation or liver damage occurs. NAFL generally does not progress to cause liver damage or complications. NASH is a chronic and progressive liver disease which can progress to cirrhosis, liver failure, and rarely liver cancer. NASH is characterized by the presence of >5% HS with inflammation and hepatocyte injury (ballooning) with or without fibrosis. According to the latest epidemiological survey, NAFLD will soon surpass chronic hepatitis virus infection as the primary cause of liver transplantation (Sayiner et al., 2016). Due to the lack of effective therapies for NAFLD, costs of care and management of associated symptoms come with a sizable economic burden (Allen et al., 2018; Estes et al., 2018).

Hepatic stellate cells (HSCs) comprise approximately 1.4% of the total liver volume and represent 5–8% of all liver cells. HSCs are typically located in the perisinusoidal space of Disse, a recess between endothelial cells of sinusoids and hepatocytes (Tacke and Weiskirchen, 2014). Upon liver injury, HSCs are activated, lose lipid-rich granules, and are transdifferentiated into a-smooth muscle actin (a-SMA)-positive myofibroblasts, which produce increased amount of ECM, proinflammatory, and profibrogenic cytokines, and cause liver fibrosis (Kamm and McCommis, 2022).

As a result, the role of HSC activation in liver fibrosis has been widely accepted and draws much attention. However, according to the view in the current studies, HSCs appear to respond to inflammatory signaling from the sinusoids. Whether and how HSCs participate in hepatic inflammation have not been examined in NAFLD. HSCs from both humans and rodents produce cytokines and chemokines upon aberrant stimuli such as lipopolysaccharide (LPS) and other toxic substances, suggesting that HSCs can potentially regulate hepatic immune and inflammatory responses through their own gene expression (Fujita and Narumiya, 2016). However, whether HSCs take part in the development of liver inflammation, and if so, whether they take on pro- or anti-inflammatory roles, is still controversial (Fujita and Narumiya, 2016). We will review the pivotal effects and mechanisms of HSCs from the perspective of inflammation and fibrosis, and highlight the potential value of HSC-targeted treatment methods in NAFLD.

## Natural history and pathogenesis of NAFLD

Clinically, NAFLD develops in four main stages (Powell et al., 2021). The main stages of NAFLD are: 1) Fat accumulation in the liver cells (steatosis), largely harmless, may be detected only during tests for another reason and this period tends to be termed as NAFL stage. 2) NASH, a more serious form of NAFLD, in which the liver has become inflamed. 3) Fibrosis, which causes scarring around the liver and nearby blood vessels, but the liver is still able to function normally. 4) Cirrhosis is the most advanced stage of the disease, caused by years of inflammation, in which the liver shrinks and becomes scarred and lumpy. It can take many years for fibrosis or cirrhosis to cause permanent damage to the liver, which can lead to liver failure or liver cancer. The majority of people only experience the first stage, which is often not noticed. In a few cases, however, the disease can progress and lead to liver damage if it is not treated. The natural history of patients with NAFLD has been studied for the past several decades (Cohen et al., 2011). Those with histological NASH, especially with some degree of fibrosis, are at a higher risk of suffering adverse outcomes such as cirrhosis and death from liver disease. The prevalence of NASH in NAFLD is estimated at 20%, and up to 20% of these patients may develop cirrhosis over the next 3–4 decades (Singh et al., 2015). Therefore, HCC screening and surveillance is routinely recommended in patients with NASH-related cirrhosis by many guidelines and associations.

NAFLD is primarily caused by overnutrition, which causes the accumulation of ectopic fat and expansion of adipose deposits. NAFLD is often associated with adipocyte enlargement and consequent macrophage recruitment and inflammation. The activation of macrophages in the visceral adipose tissue compartment creates an inflammatory state that leads to insulin resistance. Due to insulin resistance, lipolysis results in fatty acids being unabatedly delivered to the liver, which, combined with increased *de novo* lipogenesis, overwhelms its metabolic capabilities. When lipid metabolism is skewed, lipotoxic lipids can form, leading to cellular stress, activation of the inflammasomes, and apoptotic cell death. This promotes inflammation, tissue regeneration, and fibrogenesis in the body (Powell et al., 2021). In recent years, the “two-hit” hypothesis has been put forward as an explanation of NASH pathogenesis (Tariq et al., 2014). Based on this theory, NASH is not developed solely as a result of steatosis, but from an additional hit due to other factors including various cellular stress as mentioned previously. Nevertheless, this viewpoint is considered outdated now. NASH is a complex disease with many molecular pathways contributing to its development, and it is still unclear whether NASH is always preceded by NAFL. In addition, pathogenic drivers are unlikely to be identical across all patients. Therefore, both pathogenesis of disease and its clinical manifestations are highly heterogeneous (Friedman et al., 2018). There is now a widely accepted theory known as “multiple-hit,” in which genetics and environment combine to cause more widespread metabolic dysfunction as well as changes in cross-talk among several organs and tissues, including adipose tissue, the pancreas, the gut, and the liver (Fang et al., 2018). In the pathogenesis of NAFLD and its progression, several questions remain unclear. The initial “two-hit” theory cannot explain the pathogenesis of NAFLD in its entirety as it involves multiple factors. Nevertheless, fibrosis in the liver is mediated by the activation of HSCs and release of extracellular matrix (ECM) associated with hepatocyte injury and inflammation. A driving force behind hepatic fibrosis is inflammation in the liver. Also, the subsequent deleterious effects and mechanisms are worth more investigation in the pathogenesis of NAFLD.

## Inflammation and fibrosis in NAFLD

NASH, a progressive form of NAFLD, is characterized by liver steatosis, inflammation, hepatocellular injury, and fibrosis of different degrees. Also, NASH, characterized by the presence of 5% steatosis and inflammation with hepatocyte injury, has an increased liver-related mortality rate according to relevant studies (GBD 2015 Mortality and Causes of Death Collaborators, 2016; Sayiner et al., 2016). As a result of hepatocyte injury followed by inflammation, activation of the innate immune system, and release of extracellular matrix, liver fibrosis is induced. Therefore, understanding these mechanisms of inflammation and fibrosis will enable the design of targeted therapies that can halt or reverse NAFLD progression.

**FIGURE 1:**
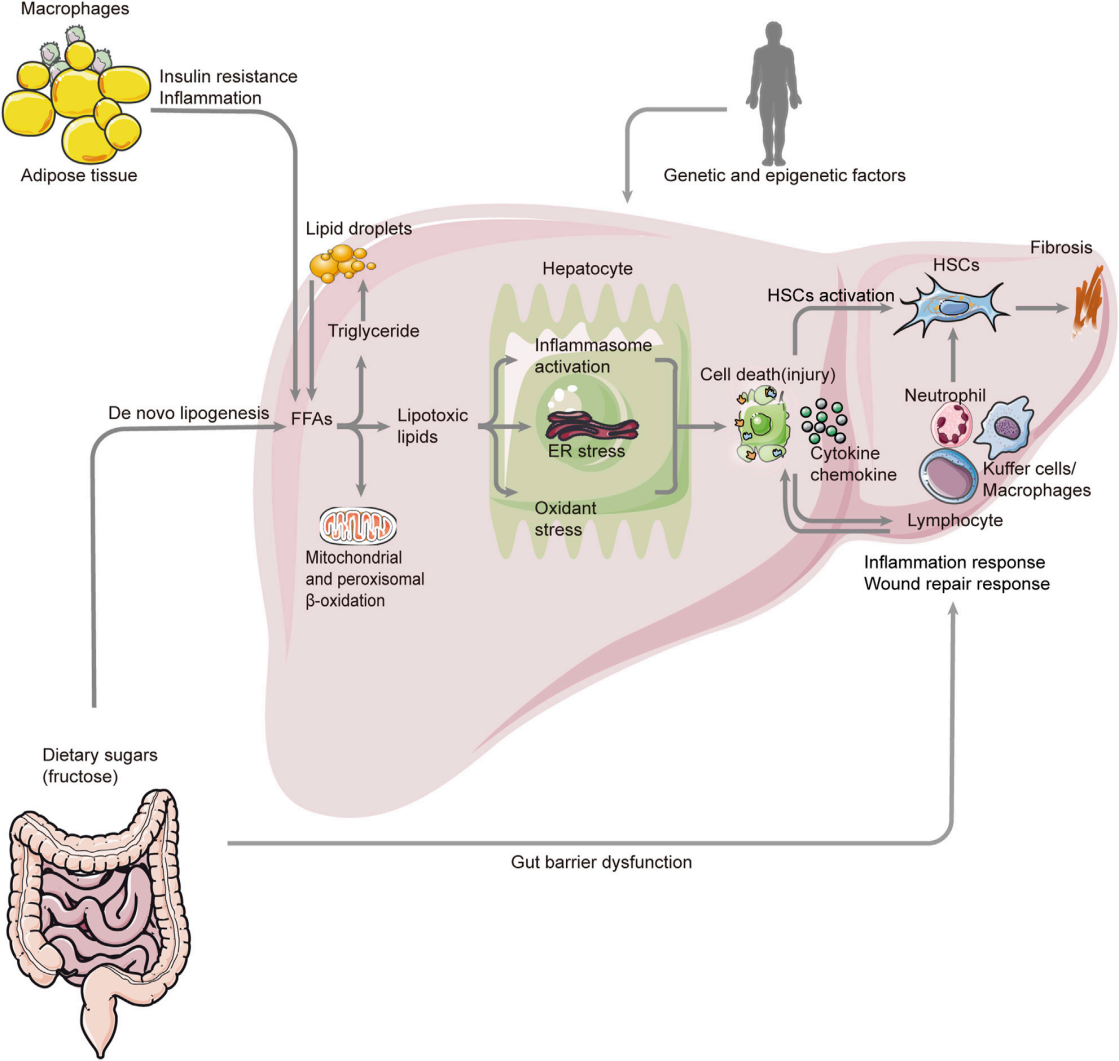
Overview pathogenesis of NASH. The pathogenesis of NASH involves many aspects such as genetic and epigenetic factors, metabolism, gut–liver axis, cell death (injury), inflammation, fibrosis, and so on. Also, the FAA is a key factor in this process. There are two major sources of FAAs. One is from the lipolysis of triglyceride in adipose tissue, and the other one is *de novo* lipogenesis. The excessive accumulation of FAAs and triglyceride leads to lipogenesis, which triggers inflammasome activation, ER stress, and oxidant stress. These result in the death (injury) of hepatocytes and the release of chemokines and cytokines. Also, they activate HSCs, which can cause fibrosis. At the same time, many immune cells respond to this process, promoting inflammation and fibrosis. In addition, the altered gut microbiome and permeability can also cause inflammation response by activating the inflammatory cells. NASH: non-alcoholic steatohepatitis; FAA: free fatty acid; ER: endoplasmic reticulum; HSC: hepatic stellate cell.

Prior to all symptoms, inflammation is a key pathophysiological mechanism and a target for therapeutic intervention. In view of the most obvious difference between NAFL and NASH, the key issue in this area is the identification of those factors that cause inflammation, thus fueling the transition from simple fatty liver to inflammatory fatty liver (see Figure 1). Since the liver is responsible for the metabolism of lipids and glucose, liver inflammation is closely associated with metabolic disorders such as NAFLD. *De novo* lipogenesis, reduced B oxidation, and a decreased VLDL cause steatosis in liver cells. Steatosis is characterized by the accumulation of triglycerides (TGs) as a compensatory response to an increase in free fatty acids (FFAs) within the cell. Moreover, the FFAs are partitioned into an inert and stable lipid, preventing the injury of hepatocytes (Kawano and Cohen, 2013; Branković et al., 2022). As a matter of fact, there have been abundant reviews of the mechanisms underlying steatosis development elsewhere. Hence, this article focuses on factors that promote and sustain hepatic inflammation in NAFLD, and attempts to summarize the functional links and key pathways in this process. According to previous investigations, multiple and various factors could act simultaneously, sequentially, or with different hierarchies in NAFLD over time. Also, clinical and histological characteristics of NAFLD are determined by these complex factors, which include both extrahepatic and intrahepatic effects. Therefore, the roles of these factors would be clarified from intrahepatic and extrahepatic angles. Intrahepatic factors in NAFLD encompass a variety of hepatocellular stress, hepatocellular death, nuclear receptors, and proteins expressed by the liver, innate immunity, genetics, and epigenetics of heritability (Lotze et al., 2007; Wagner et al., 2011; Wree et al., 2013; Luedde et al., 2014; Foroughi et al., 2016; Magee et al., 2016; Meex and Watt, 2017; Eslam et al., 2018), while extrahepatic factors in NASH mainly include dysregulated hepatic glucose, lipid metabolism, and the gut microbiota or the so-called gut–liver axis (Samuel and Shulman, 2012; Brandl and Schnabl, 2017; Caussy et al., 2019). In summary, the liver has to respond to a variety of signals that arrive from adipose tissue and the gastrointestinal tract. For example, as visceral adipose tissue increases, M1 macrophages are infiltrated, which exacerbates insulin resistance and adipose tissue inflammation and causes an altered adipokine profile. As a result of adipose tissue lipolysis, visceral adipose tissue macrophages secrete chemokines and cytokines that induce liver inflammation and IR. Increased FFA levels in the blood cause hepatic lipotoxicity and cell death. Dietary factors can exacerbate inflammation in the liver through a variety of mechanisms, including lipotoxic effects, mitochondrial dysfunction, oxidative stress, and apoptosis. The loss of the gut barrier leads to a higher bacterial translocation and circulating levels of pathogen- or microorganism-associated molecular patterns (P/MAMPs) that can initiate an inflammatory response. The risk of developing NASH is also determined by genetic and epigenetic factors. Inflammation can be resolved by a shift from M2 macrophages to specialized proresolving mediators. The transitions from NAFLD to NASH can be triggered by intrahepatic or extrahepatic factors. Moreover, a variety of inflammatory cells, including hepatic macrophages, T- and B-lymphocytes, NK cells, and platelets, as well as key effectors, such as cytokines, chemokines, and damage-associated molecular patterns, also participate in the process of inflammation in NAFLD. Due to their complex links to HSCs and key pathways related to fibrosis, these significant topics would be discussed later (Schuster et al., 2018).

It is also worth noting that NASH is characterized histologically by fibrosis as the only predictor of clinical outcome (Dulai et al., 2017; Sanyal et al., 2019). As mentioned previously, fibrosis in the liver is mediated by the activation of hepatic stellate cells in response to hepatocyte injury, inflammation, and activation of the innate immune system. The accumulation of extracellular matrix in the liver is the main cause of liver failure and death in patients with NAFLD due to fibrosis and cirrhosis. Fibrogenesis is triggered by signaling from stressed or injured hepatocytes as well as activated macrophages. This causes resident HSCs to become myofibroblasts to produce matrix proteins faster than they are degraded. HSCs are activated as a result of the inflammatory activity of liver immune cells, predominantly macrophages. TGF-B1 derived from macrophages triggers the activation of fibroblasts and is the strongest fibrogenic stimulant known (Hellerbrand et al., 1999). Furthermore, macrophages in the liver, including Kupffer cells and recruited macrophages, are capable of enhancing liver fibrosis by promoting the survival of HSCs. Then, it can be obviously considered that the cellular source of fibrosis in NASH is the hepatic stellate cell (Tsuchida and Friedman, 2017). Therefore, it is necessary to investigate the unique roles of HSCs in NAFLD from the perspective of inflammation and fibrosis.

## Roles of hepatic stellate cells in NAFLD: Crosstalk between inflammation and fibrosis

Although the three-step process of “inflammation-fibrosis-carcinoma” in NAFLD has not been investigated clearly, the significance of inflammation and fibrosis can never be ignored at any time. As the foregoing, a variety of cell types including hepatocytes, sinusoidal endothelial cells, inflammatory cells, and hepatic stellate cells participate in the process of deterioration from NAFL to NASH and even cirrhosis and liver cancer. Based on this, HSCs have pivotal effects in NAFLD and relevant mechanisms could help clarify the intricate cross-talk between inflammation and fibrosis.

As a subpopulation of liver cells, HSCs perform numerous critical functions in a healthy liver and in response to injury. Hepatic stellate cells maintain a quiescent state in which they store retinoids and vitamin A-containing metabolites in the absence of liver injury. Some less-appreciated functions of HSCs include amplifying the inflammatory response to liver injury, releasing growth factors that are critical for liver development, and liver regeneration. Therefore, HSCs could be classified into two subtypes, the quiescent HSCs and activated HSCs according to their functional statues (Kamm and McCommis, 2022). However, once HSCs were activated by liver injury such as NASH, differentiation from adipogenic to myofibroblast phenotype with the loss of lipid droplets and expression of contractile fibers, increased cell proliferation, increased HSC chemo taxis as well as signaling to attract leukocytes, and development of matured rough endoplasmic reticulum to support the production of extracellular matrix fibers and matrix remodeling enzymes would be detected from HSCs. While this review does not focus on HSC activation and fibrogenesis, there are many classic reviews available elsewhere on this topic (Friedman, 2000; Bataller and Brenner, 2005; Fujita and Narumiya, 2016; Zisser et al., 2021). Alternately, investigating the unique roles of HSCs in NAFLD is necessary. HSC activation and fibrosis are considered key factors that lead to NAFLD progression. Numerous mechanisms have been proposed for the progression of simple steatosis into NASH and NASH-associated fibrosis. From liver inflammation to fibrosis, HSCs could be triggered by numerous intrahepatic and extrahepatic factors produced by the body immune system through inflammatory response. Quiescent HSCs are located in the space of Disse, a perisinusoidal space between hepatocytes and liver sinusoidal endothelial cells, storing vitamin A in lipid droplets, which represent the major vitamin A storage site for humans (Bataller and Brenner, 2005). PPARy, adiponectin receptor 1, perilipin 2 (PLIN2/ADFP), C/EBPS, C/EBPB, and SREBP-1c are all expressed by HSCs (She et al., 2005; Kisseleva et al., 2012). Moreover, activated HSCs are a major source of ECM production in liver fibrosis caused by NASH, and are also responsible for liver inflammation induced by cytokines. On one hand, the activation of HSCs results from inflammatory activity of liver immune cells, which has been introduced earlier. The activation of HSCs in obesity is also mediated by leptin, an adipocyte-derived hormone (Potter et al., 2003). Through portal circulation, gut microbes easily enter the liver, activating toll-like receptors (TLRs) in liver cells, TLR4 in particular (Ganz and Szabo, 2013). Numerous reports have highlighted the importance of TLR4, which is a receptor for LPS. Serum levels of LPS are elevated in patients with NASH and animal models of NASH, suggesting that TLR4 is activated (Cani et al., 2007; Miele et al., 2009). The downregulation of transforming growth factor (TGF)-B pseudoreceptor BAMBI also occurs in HSCs by TLR4 ligation, resulting in increased expression of ECM by HSCs (Seki et al., 2007). What is noteworthy is that activated HSCs can also contribute to liver inflammation by secreting cytokines (Wang et al., 2017; Schuster et al., 2018). According to several research studies, HSCs can express multiple toll-like receptors which could also be induced by inflammation (Peverill et al., 2014; Seki and Schwabe, 2015). According to research on the transcription regulation of NASH, it is also reported that the transcriptional dynamics of HSCs are highly similar across diseases, suggesting HSC activation as a point of convergence during the development of NASH (Marcher et al., 2019). Also, this activation could induce the effects on inflammation by the transcriptional regulators ETS1 and RUNX1. Another research study found that HSCs could be regulated by the CD8^+^ T cells and promote the proliferation of this kind of immune cells, which were highly associated with inflammation in the liver of NASH patient (Breuer et al., 2020).

**FIGURE 2:**
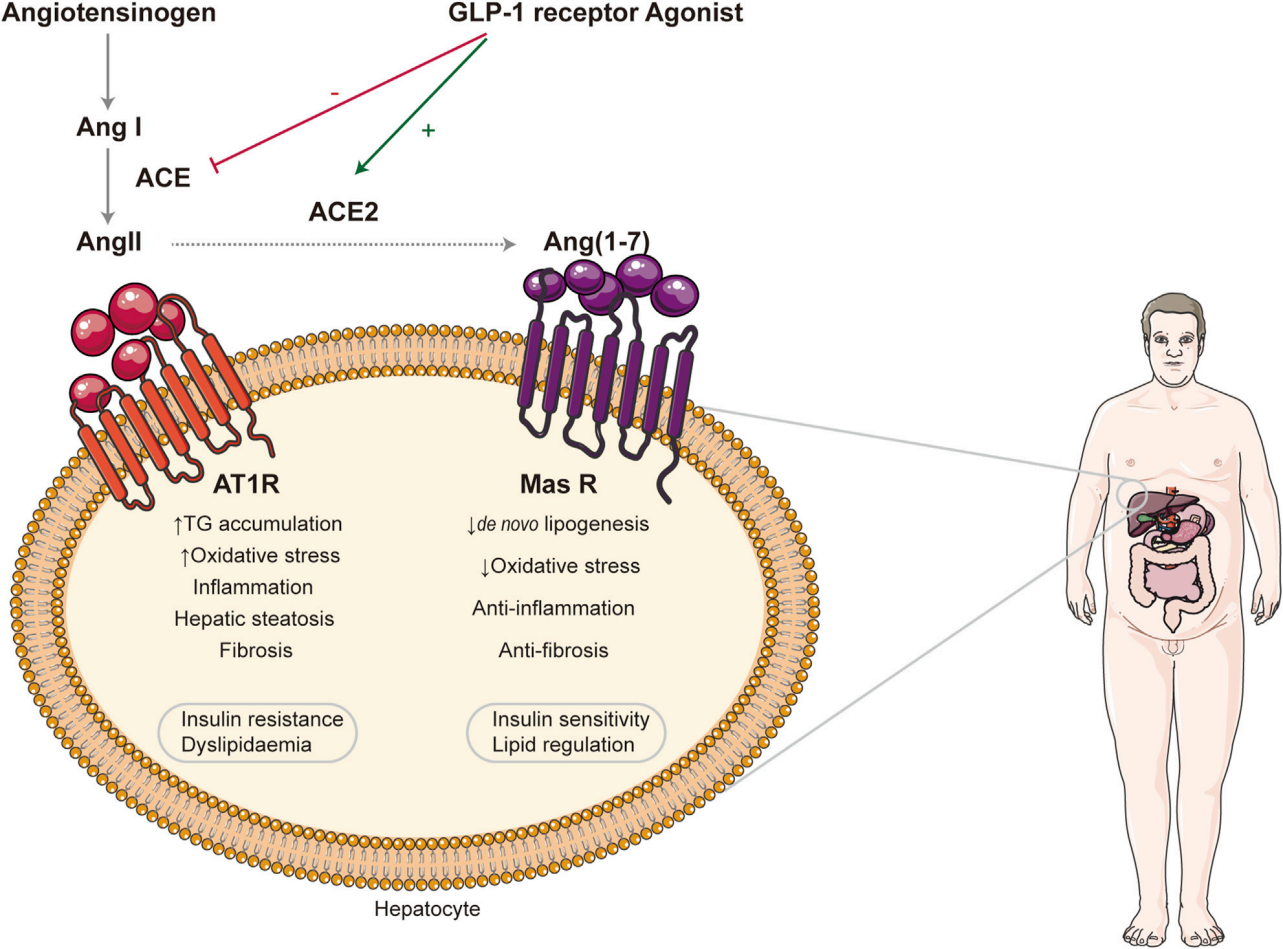
Effect of renin angiotensin systems in NAFLD. Two RAS axes, ACE/AngII/AT1R and ACE2/Ang (1–7)/Mas R, are illustrated. The former can promote the progression of NAFLD, while the latter plays the opposite role. GLP-1 receptor agonist exerts its therapeutic effect by regulating the balance between them. RAS: renin angiotensin systems; ACE: angiotensin-converting enzyme; AngII: angiotensin II; AT1R: angiotensin II type1 receptor; Ang (1–7): angiotensin1-7; Mas R: Mas receptor; GLP-1: glucagon-like peptide 1.

Renin angiotensin system (RAS) is an essential hormone system that regulates blood pressure and maintain fluid homeostasis (Simões et al., 2017). Also, the components of RAS are present locally in the liver as well. It has been reported that NAFLD has been linked to the activation of RAS both in circulation and locally in the liver (Vergniol et al., 2011; Yang et al., 2020). RAS involved two pathways: one is the classical pathway, which consists of angiotensin converting enzyme (ACE)—angiotensin Ⅱ (Ang Ⅱ)—angiotensin Ⅱ type1 receptor (AT1R), ACE/AngII/AT1R. ACE plays a crucial role in the classic RAS pathway, in which it converts AngI into AngII, and AngII mediates its biological effects mainly through receptor type 1 (AT1). It has been proved that the activation of this cascade can upregulate the level of TNF-αand TGF-β, which can activate HSC by paracrine and promote the development of NAFLD (Browning and Horton, 2004). In addition, large amounts of AngII are known to increase intracellular ROS leading to the accumulation of lipid peroxidation end-product aldehydes, such as MDA and 4-HNE, which can also convert qHSC into aHSC. Research studies indicated that activated HSCs highly expressed ACE and AT1R in the liver injuries both *in vivo* and vitro (Wei et al., 2009; Shim et al., 2018). Apart from that, angiotensin converting enzyme2 (ACE2), angiotensin1-7 [Ang (1–7)], Mas receptor (MasR) constitute the ACE2/Ang-l-7/MasR axis. ACE2, a homologue of ACE, has the opposite effect of ACE. It can inhabit the synthesis and the bioavailability of AngII, increase its degradation, and form Ang-l-7, which binds to Mas receptors playing the antagonistic roles of the ANG II/AT1 signaling axis, such as actively reducing blood pressure, inhibiting inflammation, proliferation, and fibrosis. It has been proved that Ang (1–7) reduced HSC activation associated with downregulating TGF-β (Rajapaksha et al., 2019; Yang et al., 2020). Thus, the balance between ACE and ACE2 is of considerable importance for homeostasis in the body, especially the development of NAFLD (see Figure 2). Glucagon-like peptide 1 (GLP-1) receptor agonist has dual effects on two axis of RAS, countering ACE/AngII/AT1R axis while activating ACE2/Ang1-7/Mas axis. This suggested potential therapeutic effect in NAFLD. Semaglutide and liraglutide, both GLP-1 receptor agonists, are being tested in the treatment of NAFLD (Potts et al., 2015; Htike et al., 2017; Newsome et al., 2021). Tirzepatide and BI456906 (ClinicalTrials.gov identifier: NCT04771273), dual glucose-dependent insulinotropic polypeptide (GIP) receptor, and GLP-1 receptors agonist compare favorably with the existing GLP-1 receptor agonists in terms of controlling blood sugar levels and weight (Hartman et al., 2020). Some clinicians also indicate that angiotensin-converting enzyme inhibitors (ACEIs) and angiotensin receptor blockers (ARBs) can delay NAFLD progression (Toblli et al., 2008). ACE inhibitor enalapril and AT1R inhibitor losartan can alleviate liver fibrosis in NAFLD patients with hypertension by inducing the activation of HSC (Yoshiji et al., 2009; Goh et al., 2015). In addition, providing exogenous ACE2 or increasing its endogenous expression may serve as new targets for NAFLD. Oral treatment with Ang1-7 improves inhibiting liver inflammation in high-fat diet-fed rats (Santos et al., 2013).

In short, the progression of liver fibrosis involves multiple events, of which the activation of HSCs is publicly recognized as the central element. Activated HSCs respond to the stimulus from the intracellular and extracellular microenvironment by interacting with other cells, thereby triggering wound-healing. Through diverse molecular mechanisms and cell signaling, “responded” cells play dual roles in the activation of HSCs during different stages of fibrosis.

## Perspectives of pharmacotherapies on HSCs

Since the mechanism underlying the NAFLD, which is a complicated and intricate process involving numerous risk factors, kinds of organs, different cell types, and the interplay of multiple signaling pathways, (Tilg and Moschen, 2010), has not been fully elucidated, there is no unified, clear, effective treatment for it currently. Lifestyle modification by dietary caloric restriction, exercise, and weight loss is the primary therapy for NAFLD. A loss of 5–7% of the initial weight within one year for patients with NAFLD is recommended, while in patients with suspected NASH, the targeted needs to be increased to 7–10%. Fibrosis may be attenuated only in patients with a weight loss of ≥10% (Vilar-Gomez et al., 2015; Romero-Gómez et al., 2017). Unfortunately, most patients fail to meet the goal and benefit from lifestyle intervention due to the lack of compliance and poor self-control (Vilar-Gomez et al., 2015). Surgical treatment for NAFLD mainly includes bariatric surgery, also called metabolic surgery, and liver transplantation. Studies have showed that bariatric surgery can not only improve the histological features of NASH with fibrosis or compensated cirrhosis but also reduced the risk of cardiovascular events effectively (Maciejewski et al., 2016; Ikramuddin et al., 2018; Aminian et al., 2019). As for liver transplantation, it remains the only possible intervention in end-stage liver disease or hepatocellular carcinoma related to NASH (Chalasani et al., 2018). But considering the high recurrence rate, prohibitive risk, and high cost, the application of the surgery has been limited clinically.

**FIGURE 3:**
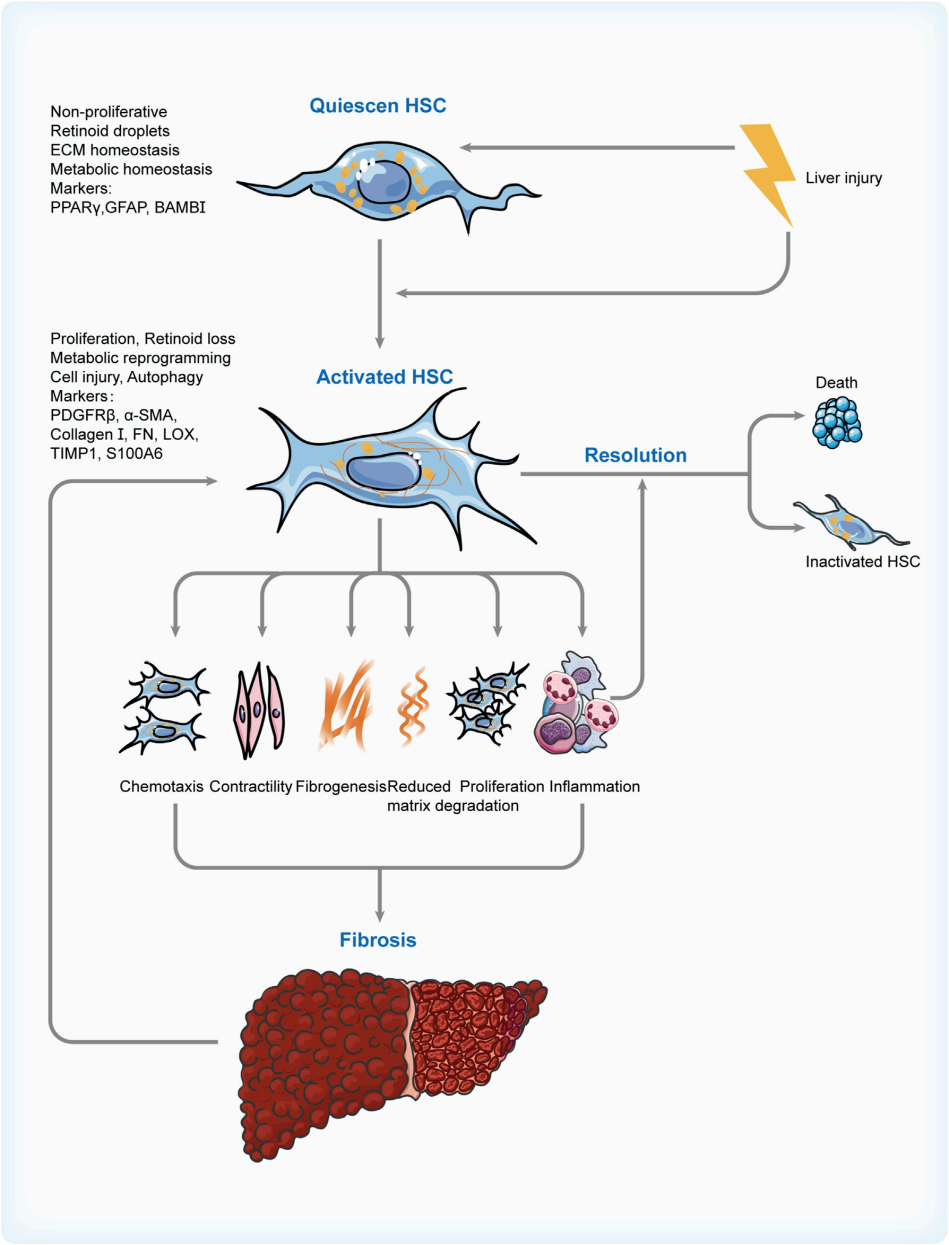
Switch of HSC phenotypes in NAFLD. Risk factors involved in the progression of NAFLD activate the HSCs and cause a sequence of changes in their features, phenotypes, and functions. These contribute to fibrogenesis and inflammation in the liver, which can promote the activation of HSCs in turn and create a vicious cycle. Thus, regulating the apoptosis of activated HSC and its reversion to inactivated phenotype through various means is the critical resolution of NAFLD. HSC: hepatic stellate cell; NAFLD: non-alcoholic fatty liver disease; PPARγ: peroxisome proliferator-activated receptor γ; GFAP: glial fibrillary acidic protein; BAMBI: bone morphogenetic protein and activin membrane bound inhibitor; PDGFRβ: platelet-derived growth factor receptor β; αSMA: α-smooth muscle actin; FN: fibronectin; LOX: lysyl oxidase; TIMP1: tissue inhibitor of metalloproteinase 1; S100A6: S100 calcium-binding protein A6.

Taking all these into consideration, clinically effective and safe pharmacological treatments are in great demand (see Figure 3). Medication development should be based on drug targets and the mechanism of action. Given the significant role of HCSs in this pathological process from the perspective of inflammation and fibrosis, the medical therapies for NAFLD associated with HSCs are focused on here (see Table 1).

**TABLE 1:** Clinical trials of medications associated with HSCs for NASH.

Medication	Primary mechanism	Trial number	Major inclusion criteria	Outcome(s)	Status
Pioglitazone	PPARδ agonist	NCT00063622	Patients with NASH and without diabetes	No benefit of pioglitazone over placebo in fibrosis score (34% and 19%, respectively; *p* = 0.04)	Phase 3; recruitment status: completed
Pioglitazone	PPARδ agonist	NCT00227110	Patients with impaired glucose tolerance or type 2 diabetes and liver biopsy-confirmed NASH	Improvement in metabolism and histological features	Phase 4; recruitment status: completed
Elafibranor	PPARα and PPARβ/δ agonist	NCT02704403	Patients with NASH and fibrosis	No significant difference in the improvement of fibrosis between treatment and placebo groups	Phase 3; recruitment status: terminated (study did not meet the predefined primary surrogate efficacy endpoint, no safety issues identified)
Lanifibranor	PPARα,PPARγ and PPARβ/δ agonist	NCT03008070	Patients with noncirrhotic, highly active NASH	Significant decrease of at least two points in the SAF-A score without worsening of fibrosis with the 1200-mg dose of lanifibranor than with placebo	Phase 2; recruitment status: completed
Selonsertib (STELLAR 3)	ASK-1 inhibitor	NCT03053050	Patients with NASH and bridging fibrosis (F3, STELLAR-3)	No antifibrotic effect	Phase 3; recruitment status: terminated (this study was terminated early due to the lack of efficacy based on the results of the week 48 analysis as prespecified in the clinical study protocol.)
Selonsertib (STELLAR 4)	ASK-1 inhibitor	NCT03053063	Adults with compensated cirrhosis (F4, STELLAR-4) due to NASH	No antifibrotic effect	Phase 3; recruitment status: terminated (this study was terminated early due to the lack of efficacy based on the results of the week 48 analysis as prespecified in the clinical study protocol.)
Pentoxifylline	PDE4 inhibitor	NCT04977661	Egyptian NASH patients	Safe and effective in improving liver aminotransferases, serum cytokine, and chemokine in Egyptian NASH patients	Phase 4; recruitment status: completed
ASP9831	PDE4 inhibitor	NCT00668070	NASH and elevated serum ALT levels	No significant difference in altering the biochemical markers of NASH, compared to placebo	Phase 2; recruitment status: completed
Roflumilast	PDE4 inhibitor	NCT01703260	Patients with a historical diagnosis of NASH	No results available	Phase 2; recruitment status: terminated (company decision; no safety or efficacy concerns)
Emricasan	Caspase inhibitor	NCT02686762	Patients had definite NASH and NASH CRN fibrosis stage F1-F3	No improvement in liver histology in patients with NASH fibrosis and with the possibility of worsening fibrosis and ballooning	Phase 2; recruitment status: completed
Emricasan	Caspase inhibitor	NCT02960204	Patients with NASH-related cirrhosis and baseline HVPG ≥12 mmHg	No significant improvement in HVPG or clinical outcomes in patients with NASH-related cirrhosis and severe portal hypertension	Phase 2; recruitment status: completed
Emricasan	Caspase inhibitor	NCT03205345	Subjects with decompensated NASH cirrhosis	Safe but ineffective for treatment of decompensated NASH cirrhosis	Phase 2; recruitment status: unknown
GS-9450	Caspase inhibitor	NCT00740610	Patients with elevated ALT (>60 U/L at screening), fatty liver on screening ultrasound, and biopsy-proven NASH	Significant reductions in ALT levels in NASH patients with GS-9450 treatment groups	Phase 2; recruitment status: completed
Obeticholic acid (REVERSE)	FXR agonist	NCT03439254	Subjects with a confirmed diagnosis of NASH and a fibrosis score of 4 based upon the NASH CRN scoring system determined by central reading	No results available	Phase 3; recruitment status: active, not recruiting
Obeticholic acid (REGENERATE)	FXR ligand	NCT02548351	Patients with definite NASH, NAFLD activity score of at least 4, and fibrosis stages F2-F3, or F1 with at least one accompanying comorbidity	Obeticholic acid 25 mg improved fibrosis and key components of NASH disease activity among patients with NASH	Phase 3; recruitment status: active, not recruiting
Tropifexor (FLIGHT-FXR)	FXR agonist	NCT02855164	NASH on biopsy with stage 1–3 fibrosis or clinical risks for NASH	Anti-inflammatory and anti-steatogenic effects were observed in part A/B of a phase 2 trial. A significant reduction in ALT levels, weight loss, and liver fat loss according to the interim analysis in Part C. Adverse effects: pruritus, elevated LDL and decreased HDL	Phase 2; recruitment status: terminated
Cenicriviroc	CCR2/CCR5 inhibitor	NCT03028740	Subjects with NASH and stage F2 or F3 fibrosis	No results available	Phase 3; recruitment status: terminated (This study was terminated early due to lack of efficacy based on the results of the planned interim analysis of part 1 data.)
Simtuzumab (SIM, GS-6624)	LOXL2 inhibitor	NCT01672879	Patients with bridging fibrosis caused by NASH	Ineffective in decreasing hepatic collagen content or HVPG	Phase 2; recruitment status: terminated
		NCT01672866			
ND-L02-s0201 Injection	HSP47 inhibitor	NCT02227459	Moderate to extensive hepatic fibrosis (METAVIR F3-4)	No results available	Phase 1; recruitment status: completed
IMM-124E	Anti-LPS	NCT02316717	Diagnosis of NASH	No significant improvement in clinical endpoints	Phase 2; recruitment status: completed
Liraglutide	GLP-1 agonist	NCT02654665	Obese adults with non-alcoholic fatty liver disease	Effective for decreasing weight, hepatic steatosis, and hepatocellular apoptosis in obese adults with NAFLD	Phase 3; recruitment status was: recruiting
Semaglutide	GLP-1 agonist	NCT02970942	NASH on biopsy with stage 1–3 fibrosis	Significant improved NASH, but no improvement in fibrosis	Phase 3; recruitment status: completed
Tirzepatide	Dual GIP-GLP1 agonist	NCT04166773	Diagnosis of NASH	No results available	Phase 2; recruitment Status: recruiting
BI456906	Dual GIP-GLP1 agonist	NCT04771273	Diagnosis NASH and fibrosis stage F1-F3	No results available	Phase 2; recruitment Status: recruiting

HSC, hepatic stellate cell; NASH, non-alcoholic steato hepatitis; PPAR, peroxisome proliferator-activated receptor; SAF-A score, the activity part of the steatosis, activity, and fibrosis [SAF] scoring system that incorporates scores for ballooning and inflammation; ASK-1, apoptosis signaling kinase-1; PDE4, cyclic nucleotide phosphodiesterase 4; ALT, alanine aminotransferase; CRN, clinical research network; HVPG, hepatic venous pressure gradient; FXR, Farnesoid X receptor; NAFLD, non-alcoholic fatty liver disease; CCR2/CCR5, the C–C motif chemokine receptor 2 (CCR2)–CCR5; LOXL2, lysyl oxidase-2; HSP47, heat shock protein 47; LPS, lipopolysaccharide; GLP-1, glucagon-like peptide 1; GIP, glucose-dependent insulinotropic polypeptide.

Peroxisome proliferator-activated receptor (PPAR) belongs to nuclear receptor superfamily and comprises three forms: PPARα, PPAβ/δ, and PPARγ involving in modulating lipid metabolic homeostasis (Oh and Ahn, 2021). It has been introduced that PPARs can modulate HSC activation though defending against lipid-related toxicity and mitigate hepatocyte stress (Bojic and Huff, 2013; Pawlak et al., 2015). However, clinical studies are yet to validate their effects on fibrosis related to NASH. Pioglitazone, a PPARγ-specific agonist; elafibranor, a dual PPARα/δ agonist; and lanifibranor, a PPARα/δ/γ/ are all under clinical trials (Sanyal et al., 2010; Ratziu et al., 2016; Francque et al., 2021).

As is known, hepatocyte apoptosis aggravates inflammatory response, and apoptotic bodies can activate HSCs and promote their transition to myofibroblasts, which enhance fibrogenesis (Canbay et al., 2003). Apoptosis signa-regulating kinase 1 (ASK-1), a member of MAP kinases (MAP3K), is expressed by inflammation and HSCs. Also, it can cause apoptosis and increase the expression of the proinflammatory and profibrotic cytokine in response to various stressors in turn (Schuster and Feldstein, 2017). Therefore, the inhibitor of ASK1—selonsertib, can ameliorate NASH in some patients in a 24-week clinical trial (Loomba et al., 2018). Nevertheless, the outcomes are not satisfied in phase 3 trials (Harrison et al., 2020a). Emricasan, a caspase inhibitor, has the effects similar to those of selonsertib (Harrison et al., 2020b; Garcia-Tsao et al., 2020).

Pentoxifylline (PTX), a cyclic nucleotide phosphodiesterase 4 (PDE4) inhibitor, had been proven in numerous research studies to inhibit the proliferation and activation of HSCs either by direct or indirect ways (Desmoulière et al., 1999; Gonzalo et al., 2006; Verma-Gandhu et al., 2007). PTX improved hepatic aminotransferases and inflammatory markers in Egyptian NASH patients in the clinical trials (Fouda et al., 2021). In addition, ASP9831 and roflumilast both belong to the PDE4 inhibitor.

C-C motif chemokine receptors 2 and 5 (CCR2 and CCR5) can promote the activation and migration of Kupffer cells and HSCs and increase inflammatory cells (Friedman et al., 2016), and thus the dual CCR2/CCR5 inhibitor CVC can reduce fibrosis progression to some extent, which was evident in animal models (Lefebvre et al., 2016). Unfortunately, it showed no significant effect in phase 3 clinical trial (Anstee et al., 2020).

Farnesol X receptor (FXR) plays a vital role in regulating cholesterol and bile acid metabolism, and it can be expressed by HSCs and other kinds of cells associated with enterohepatic circulation. The activating FXR inhibited hepatic inflammation and fibrosis (Verbeke et al., 2016). Obeticholic acid (OCA), an FXR agonist, prevents the activation of HSCs and reduces the fiber generates related to protein, such as Col1a1, α-smooth muscle actin, and tissue inhibitors of metalloproteinases-1, -2, significantly contributing to the improvement in liver fibrosis (Zhou et al., 2020). Phase 3 clinical trials confirmed this positive effect on fibrosis, while accompanied with adverse effects including pruritus and elevation of low-density lipoprotein (Younossi et al., 2019). Tropifexor is another FXR agonist under reaserch.

Activated HSCs express high level of lysyl oxidase-like 2 (LOXL2) (Brovold et al., 2020), a key stromal enzyme in collagen formation. It can modify the extracellular matrix by promoting the cross-linking of collagen fibers resulting in the exacerbation of fibrosis, which can activate HSCs in turn. The inhibitory antibody against LOXL2, simtuzumab (previously known as GS-6624), was expected to mitigate this vicious circle in NASH. But it failed to perform well clinically (Harrison et al., 2018). Heat shock protein 47 (HSP47), a chaperone protein, is involved in regulating the folding of fibrillary collagens. Inhibiting this molecule causes collagen to misfold within the cell and to accumulate rather than secrete, resulting in HSC death eventually. Currently, a trial of liposomal formulation is under way in patients with advanced fibrosis. This study involved silencing HSP47 by siRNA to promote the apoptosis of HSCs (NCT02227459).

In addition, targeting intestinal lipopolysaccharide (LPS) can also obtain the anti-inflammatory and antifibrotic effects, for instance, IMM124E (Mizrahi et al., 2012). Although many pharmacotherapies on HSCs for NAFLD patients are introduced, most of them are only in the clinical trial stage, and their safety and efficacy need to be further demonstrated. Given these, there are still no specific agents that are approved by the Food and Drug Administration (FDA) currently (Sheka et al., 2020). Therefore, the combination of drugs with different targets and integrated treatment of traditional Chinese medicine and Western medicine will be the key direction for future investigations.

## Conclusion

Although HSCs comprise only a small proportion of hepatic cells, HSCs and their activation or inactivation appear to play a vital role in the development of NASH-induced hepatic fibrosis in NAFLD. Also, from the perspectives of inflammation and fibrosis, a deeper understanding of HSCs and the mechanisms leading to the investigation in NASH is, therefore, necessary if efforts are to identify potential HSC targets for drug development. Future studies are required to truly appreciate whether and how HSCs contribute to hepatic pathophysiology in NAFLD.

## Data Availability

The original contributions presented in the study are included in the article/Supplementary Material. Further inquiries can be directed to the corresponding authors.
